# Avian Interferon-Inducible Transmembrane Protein Family Effectively Restricts Avian Tembusu Virus Infection

**DOI:** 10.3389/fmicb.2017.00672

**Published:** 2017-04-20

**Authors:** Shilong Chen, Long Wang, Jieying Chen, Lanlan Zhang, Song Wang, Mohsan U. Goraya, Xiaojuan Chi, Yang Na, Wenhan Shao, Zhou Yang, Xiancheng Zeng, Shaoying Chen, Ji-Long Chen

**Affiliations:** ^1^Key Laboratory of Fujian-Taiwan Animal Pathogen Biology, College of Animal Sciences, Fujian Agriculture and Forestry UniversityFuzhou, China; ^2^Key Laboratory of Animal Virology, Institute of Animal Husbandry and Veterinary Medicine, Fujian Academy of Agriculture SciencesFuzhou, China; ^3^Department of Zoology, College of Life Sciences, Fujian Agriculture and Forestry UniversityFuzhou, China; ^4^CAS Key Laboratory of Pathogenic Microbiology and Immunology, Institute of Microbiology, Chinese Academy of SciencesBeijing, China

**Keywords:** Avian Tembusu virus, host innate immunity, interferon, IFITM, antiviral response

## Abstract

Avian Tembusu virus (ATMUV) is a highly pathogenic flavivirus that causes significant economic losses to the Chinese poultry industry. Our previous experiments demonstrated that ATMUV infection effectively triggered host innate immune response through MDA5 and TLR3-dependent signaling pathways. However, little information is available on the role of interferon-stimulated genes (ISGs) in defending against ATMUV infection. In this study, we found that ATMUV infection induced robust expression of type I and type III interferon (IFNs) in duck tissues. Furthermore, we observed that expression of interferon-inducible transmembrane proteins (IFITMs) was significantly upregulated in DEF and DF-1 cells after infection with ATMUV. Similar results were obtained from *in vivo* studies using ATMUV-infected ducklings. Importantly, we showed that knockdown of endogenous IFITM1 or IFITM3 by specific shRNA markedly enhanced ATMUV replication in DF-1 cells. However, disruption of IFITM2 expression had no obvious effect on the ATMUV replication. In addition, overexpression of chicken or duck IFITM1 and IFITM3 in DF-1 cells impaired the replication of ATMUV. Taken together, these results reveal that induced expression of avian IFITM1 and IFITM3 in response to ATMUV infection can effectively restrict the virus replication, and suggest that increasing IFITM proteins in host may be a useful strategy for control of ATMUV infection.

## Introduction

Tembusu virus (TMUV) is a member of the Ntaya virus group within the genus *Flavivirus* of family *Flaviviridae* (Yan et al., 2011). TMUV strains were firstly isolated from mosquitoes in Malaysia and Thailand, but their pathogenicity is not fully understood (Platt et al., 1975; Pandey et al., 1999). Sitiawan virus, a broiler-origin TMUV strain, was the first pathogenic virus causing encephalitis and retarded growth in broiler chicks (Kono et al., 2000). Since 2009, Chinese domestic poultry including ducks, chickens, and geese have been manifesting a new epidemic disease caused by a TMUV-related flavivirus, named as avian Tembusu virus (ATMUV). This outbreak was quickly spread to many provinces of China and several south-eastern Asian countries (Homonnay et al., 2014; Thontiravong et al., 2015). ATMUV genome consists of a single strand positive-sense RNA and encodes three structural proteins [capsid (C), pre-membrane (PrM/M), and envelope (E)] and seven non-structural proteins (NS1, NS2A, NS2B, NS3, NS4A, NS4B, and NS5) (Liu et al., 2013; Homonnay et al., 2014). ATMUV-infected adult animals showed symptoms of hemorrhagic ovaritis and acute egg drop syndrome, high fever, anorexia, diarrhea, ataxia, weight loss and paralysis, with a high morbidity (90–100%), and mortality ranged from 0 to 30% depending on different management and weather conditions, leading to enormous economic losses to poultry industry in China (Cao et al., 2011; Su et al., 2011; Yan et al., 2011; Liu et al., 2012, 2013; Chen et al., 2013). The young flocks are more vulnerable to ATMUV infection, characterized by similar clinical symptoms including anorexia, diarrhea, high fever and severe neurologic dysfunction, with a higher mortality than adult birds (Vaidya et al., 2012; Ti et al., 2015). ATMUV was easily detected in ovaries and theca folliculi of infected animals, suggesting that the reproductive tissues were the major targets for the viral infection and replication (Liu et al., 2012). Viral RNA was also detected in spleen, trachea, kidney, brain, and blood of infected host (Yan et al., 2011; Liu et al., 2012). In addition, it was observed that ATMUV could infect various cell lines including DEF, CEF, DF-1, BHK-21, Vero, A549, 293T, and HeLa, resulting in a noticeable cytopathic effect (CPE) characterized by cell shrinkage, rounding and detachment (Chen et al., 2016a; Wang H. J. et al., 2016).

It has been reported that ATMUV RNA and neutralizing antibodies was detected in duck farm workers in Shandong province of China (Tang et al., 2013), suggesting that this virus could infect human. But a disease associated with ATMUV infection has not been found in human. Moreover, it was shown that ATMUV failed to cause any clinical manifestation or viremia in non-human primates, indicating that ATMUV is unlikely to emerge as a human pathogen for the time being (Wang H. J. et al., 2016). However, due to zoonotic nature of its genus Flavivirus relatives, ATMUV might be a potential threat to human health in the future (Bowen et al., 1975; Liu et al., 2013).

Host innate immune response serves as the first line of defense against the infection of pathogens at the early stages. Innate immune system recognizes viruses invasion via specific pattern recognition receptors (PRRs) to sense pathogen associated molecular patterns (PAMPs) expressed by viruses. Activated PRRs then interact with adaptor proteins such as interferon-β promoter stimulator-1 (IPS-1), MyD88, and TRIF (Takeuchi and Akira, 2009). The ligations of PRRs with adaptor proteins result in the activation of the transcription factors, including NF-κB and interferon regulatory factors (IRF3 and IRF7) (Takeuchi and Akira, 2010). IRFs and NF-κB translocate to the nucleus where they stimulate the expression of type I and type III interferons (IFNs). Then interaction between the IFNs and their receptors causes activation of JAK-STAT signaling pathway. Phosphorylated STAT proteins translocate to the nucleus and combine with interferon regulatory proteins to promote an abundant expression of a wide array of genes, including IFN-stimulated genes (ISGs) (Takeuchi and Akira, 2010; Tan et al., 2012; Bailey et al., 2014). These ISGs encode distinct antiviral proteins with diverse biological effects that block multiple stages of the viral lifecycle including viral entry, translation, replication, assembly, and spread (Diamond and Farzan, 2013).

The interferon-inducible transmembrane proteins (IFITMs) are a family of small transmembrane proteins belonging to ISG superfamily and strongly induced by IFNs (Perreira et al., 2013). The IFITM proteins are the distinct restriction factors known to block viral entry, including restriction of virus fusion with the late endosomal or lysosomal compartments and penetration into the cytoplasm (Diamond and Farzan, 2013; Li et al., 2013). It has been shown that gene cluster IFTIM1, 2, and 3, the immune-related genes, are critically involved in immune defense against a broad variety of viruses, including influenza virus, dengue virus, filoviruses, coronavirus, hepatitis C virus, lyssaviruses, and West Nile virus (Brass et al., 2009; Huang et al., 2011; Lu et al., 2011; Smith et al., 2013; Wilkins et al., 2013). Conversely, IFITMs have little or no effect on several other viruses, including human papillomavirus, human cytomegalovirus and adenovirus type 5, arenavirus, murine leukemia virus, and foot-and-mouth disease virus (Warren et al., 2013; Bailey et al., 2014; Zhang et al., 2016). Despite the progresses in understanding of IFITM-mediated antiviral ability, host IFITM expression profiles in response to ATMUV infection are still not clear. Little is known about this immune-related ISG family in restricting ATMUV pathogenesis.

In the present study, we examined the expression of key IFNs and IFITMs in host cells after ATMUV infection *in vitro* and *in vivo*. Interestingly, we observed that ATMUV infection could trigger duck innate immune response including robust expression of particular type I and type III IFNs and IFITM family proteins. Using DF-1 cell system, we found that knockdown of endogenous IFITM1 and IFITM3 by short hairpin RNA (shRNA) markedly enhanced ATMUV infection in host. However, silencing IFITM2 had no significant effect on ATMUV replication. Furthermore, overexpression of chicken or duck IFITM1 and IFITM3 could strongly inhibit the replication of ATMUV. These results reveal that avian IFITM1 and IFITM3 but not IFITM2 serve as the critical components of host innate immune defense against ATMUV infection.

## Materials and methods

### Antibodies

The antibodies used in this study are described as follows: Mouse anti-β-actin (ab8226, Abcam, Cambridge, UK), mouse anti-Flag (HT201, TransGen, Beijing, China), HRP goat anti-mouse IgG (LP1002a, ABGENT, USA). A monoclonal antibody against E protein of ATMUV was prepared in our lab using the method described previously (Chen et al., 2008). FITC (fluorescein isothiocyanate) conjugated goat anti-mouse IgG was purchased from BOSTER (Wuhan, China). Chicken type I interferon was obtained from Dalian Sanyi Animal Medicine Co. Ltd. (Dalian, China). Lipofectamine 3000 was obtained from Invitrogen (Carlsbad, CA, USA).

### Cell lines, birds, virus, and infection

ATMUV CJD05 strain was isolated from a chicken farm with acute egg-drop syndrome in Fujian, China (Chen et al., 2013). Duck embryo fibroblasts (DEFs) were prepared from 13-day-old mule-duck embryo as previously described (Shahsavandi et al., 2013). DF-1 (immortalized chicken embryo fibroblast cell line) and 293T cells were obtained from American Type Culture Collection (Manassas, VA). DEF, DF-1, and 293T cells were cultured at 37°C with 5% CO_2_ in DMEM (Sigma, USA) supplemented with 10% fetal bovine serum (FBS, HyClone, Utah, USA), 100 units of penicillin G and 100 μg of streptomycin. DF-1 and DEF were infected with ATMUV CJD05 as previously described (Chen et al., 2016a) at the multiplicity of infection (MOI) of 1.0 and harvested at the indicated times after infection. Twenty five 7-day-old mule healthy ducklings were challenged with 0.4 mL of CJD05 (the 5th passage allantoic fluid virus, ELD_50_ = 10^−6.0^/mL) per duckling by intramuscular injection. Each group of three randomly selected ducklings was sacrificed at 0, 12, 24, 48, and 72 h post-infection (hpi), and their spleen, kidney, bursa of fabricius, pancreas, and brain were harvested for detection of viral infection by indirect immunofluorescence assay. These tissues were also used for total RNA extraction to examine the mRNA expression of IFNs/IFITMs by quantitative real-time RT-PCR (qRT-PCR). The sera and spleen homogenates (30%w/v) of the ducklings were prepared for detecting of viral titers by 50% tissue culture infectious dose (TCID_50_) assay during the infection. Other infected ducklings and 10 control ducklings (inoculated with 0.4 mL sterile PBS per duckling) were used to monitor clinical signs and rectal temperature daily for 10 days.

### Quantitative real-time RT-PCR and RT-PCR

Total RNA was extracted from the cultured cells and duckling spleen tissues using Trizol reagent (TransGen Biotech, Beijing, China) according to the manufacturer's instructions. Equal amount of RNA (4 μg) was reverse transcribed into cDNA utilizing M-MLV Reverse Transcriptase (Promega, USA). The cDNA was analyzed by qRT-PCR using TransStart Green qPCR SuperMix (TransGen) and PCR using rTaq DNA polymerase (Takara Bio). The primers specific for chicken β-actin, IFN-a, IFN-β, IFN-λ, and ATMUV E gene have been previously described (Chen et al., 2016a). The primers specific for ATMUV NS5, duck β-actin, IFN-a, IFN-β, IFN-λ, IFITM1, 2, 3, and chicken IFITM1, 2, 3 were designed using the Primer5 software (Table 1). The relative mRNA abundances were analyzed using the 2′ΔΔCt method with housekeeping gene (β-actin) as an internal normalization and plotted as fold changes compared with the mock- infected samples.

**Table 1:** **Primer sequences used in this study**.

**Primer name**	**Primer sequence (5′-3′)**
Chicken IFITM1-F	GGAGTCCCACCGTATGAAC
Chicken IFITM1-R	GGCGTCTCCACCGTCACCA
Chicken IFITM2-F	AGGTGAGCATCCCGCTGCAC
Chicken IFITM2-R	ACCGCCGAGCACCTTCCAGG
Chicken IFITM3-F	ATCGCAAAGTCCTGGGTG
Chicken IFITM3-R	TGCTGCTGGTGGTTGAAGA
Chicken IFNAR1-F	ACAGCTGGCGGTAAACACTT
Chicken IFNAR1-R	GCTAAAGAGCTGTGCTCCGA
chIFNAR1-siRNA Sense	GCAAUUUGUCAUCUGUCAUTT
chIFNAR1-siRNA Antisense	AUGACAGAUGACAAAUUGCTT
shRNA-chIFITM1	GGAGGACAGCGAAGATCTTTA
shRNA-chIFITM2	ACCATTGCCATCATGTTCATC
shRNA-chIFITM3	GCCCATCTGATCAACGTCTTC
Duck β-actin-F	CTATGTCGCCCTGGATTT
Duck β-actin-R	TAGAAGCATTTGCGGTGG
Duck IFN-α-F	TCCTGGACACCAACGACA
Duck IFN-α-R	TTGGATGCAGCCGAAGTA
Duck IFN-β-F	AACCACTACATCTACCACCTCG
Duck IFN-β-R	TCTTGCTCGGCATCCACT
Duck IFN-λ-F	CACCAGGCTCTTCAATCGGA
Duck IFN-λ-R	CAGCACTTGGAAGAGGTGGA
Duck IFITM1-F	AACCCTACGGCAGGAATG
Duck IFITM1-R	GAAGACAAGAGCGAGGAAGC
Duck IFITM2-F	GCCCGCGACTGCAAGAT
Duck IFITM2-R	GGTGATGACAGCCACGAAGA
Duck IFITM3-F	TGGCTTGGTCGCTGTGC
Duck IFITM3-R	GCAGGTTGACGACGGTGAT
ATMUV NS5-F	ACACCATTTCCACGAGC
ATMUV NS5-R	TTAGTGACCAGCCAGACC
CMV5a-chIFITM1-F	CCG*GAATTC*ATGCAGAGCTACCCTCAGCAC
CMV5a-chIFITM1-R	CGC*GGATCC*GGGCCTCACAGTGTACAACGG
CMV5a-chIFITM3-F	CCC*AAGCTT*GGGATGGAGCGGGTACGCGCTTC
CMV5a-chIFITM3-R	CGC*GGATCC*GCGAGTGGGTCCAATGAATTCGG
CMV5a- dIFITM1-F	CCG*GAATTC*ATGGAGAACTACCCGCAGTCC
CMV5a- dIFITM1-R	CGC*GGATCC*GGGGTGGTGTACTGGTCTGTA
CMV5a- dIFITM3-F	CCC*AAGCTT*GGGATGGAGCGGACCCGAGCTCCG
CMV5a- dIFITM3-R	CGC*GGATCC*GCGTGTGGGGCCGTAGAAGGG

*The Italic underlined nucleotides are restriction enzyme sites*.

### shRNA-based knockdown and generation of stable DF-1 cell lines

The specific short hairpin RNAs (shRNA) were designed for knockdown of chicken IFITM1, 2, and 3. All the shRNA sequences are shown in Table 1. Luciferase control shRNA was described previously (Wang et al., 2014). DF-1 cell lines stably expressing shRNA targeting chicken IFITMs (chIFITMs) were generated using lentiviral vectors as previously described (Wang et al., 2012). Briefly, 293T cells were cotransfected with shRNA construct and HIV-based packaging constructs (pLP, pLP1, and pLR2). Supernatant of the cultured cells containing pseudotyped lentiviruses with indicated shRNAs were collected at 48 h post-transfection and filtered through the 0.22 μM syringe-driven filter. DF-1 single-cell suspension was incubated with the supernatant and 8 μg/mL of polybrene (Sigma) and centrifuged at 2,100 rpm, 32°C for 120 min. DF-1 cells were then cultured in DMEM supplemented with 10% FBS for further studies. qRT-PCR was performed to determine the interference efficiency and mRNA expression of viral genes in DF-1 cell lines at indicated times after infection. The viral titers of supernatants were examined by TCID_50_ assay in DEF cells.

### Plasmids construction and overexpression study

Full-length cDNA encoding chicken or duck species IFITM1 and IFITM3 was subcloned into pFLAG-CMV-5a vector with a Flag tag in the COOH terminus to create DNA constructs chIFITM1, chIFITM3, duck IFITM1 (dIFITM1), and dIFITM3, respectively. The specific primers with *restriction enzyme sites* are shown in Table 1. DF-1 cells were seeded onto 6-well plates and cultured in DMEM with 10% FBS. When the cells reached 60–80% confluence, they were transfected with 3 μg of plasmid DNA/well using Lipofectamine 3000 (Invitrogen). Twenty four hours later, transfected DF-1 cells were infected with ATMUV CJD05 and harvested at indicated times (24, 36, and 48 hpi) for further qRT-PCR or Western blotting analysis. Supernatants were collected for detection of viral titers by TCID_50_ assay.

### Indirect immunofluorescence assay (IFA)

Indirect immunofluorescence assay was performed as previously described (Chen et al., 2016b). Briefly, the parenchymal organs sections and DEF cells infected with ATMUV were incubated with mouse anti-ATMUV E protein monoclonal antibody for 30 min at 37°C and then washed with PBS, followed by incubation with FITC conjugated goat anti-mouse IgG at 37°C for 30 min before imaging with fluorescence microscope (Nikon, Japan).

### Western blotting

DF-1 cells were lysed with lysis buffer containing 1 × complete protease inhibitor cocktail for 30 min on ice according to our previous method (Wang et al., 2014). Cell lysates were separated on 12% SDS-PAGE gels, transferred to PVDF membranes and blocked with 5% (w/v) milkpowder in Tris-buffered saline (pH7.4, TBS) for 2 h at room temperature. The membranes were incubated with indicated primary antibodies for 2.5 h at room temperature and washed with TBS, followed by incubation with appropriate secondary antibodies at room temperature before imaging with the ProteinSimple FluorChem M system (Bio-Techne, USA).

### Statistical analysis

Data represented the mean ± *SD*. Statistical significance was determined by one tail Student's *t*-test analysis. Differences were considered statistically significant with *P* < 0.05.

### Ethics statement

The animal protocol used in this study was approved by the Research Ethics Committee of College of Animal Science, Fujian Agriculture and Forestry University (Permit Number PZCASFAFU2014002). The procedures were carried out in accordance with the approved guidelines.

## Results

### ATMUV inoculated mule ducklings developed mild clinical symptoms and viremia

**Figure 1:**
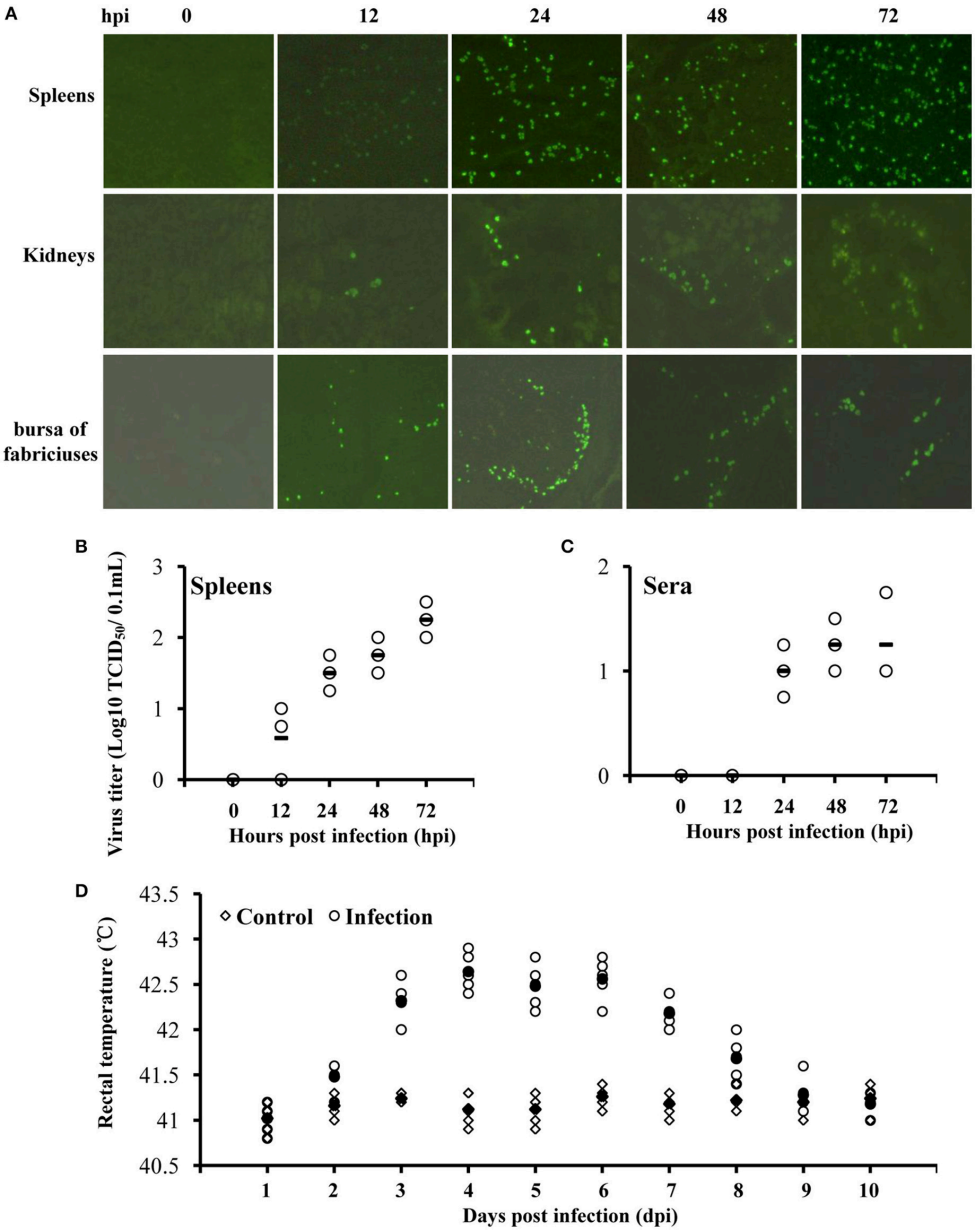
**ATMUV exhibits mild pathogenicity in mule ducklings following intramuscular injection**. Twenty five 7-day-old healthy mule ducklings were challenged by intramuscular injection with 4.0 × 10^5^ ELD_50_ of ATMUV in a volume of 0.4 mL per duckling. Ten same old ducklings were inoculated with 0.4 mL sterile PBS per duckling as a control. Three randomly selected ducklings were sacrificed at 0, 12, 24, 48, and 72 hpi, respectively. **(A)** Their organ tissues were harvested for detection viral infection by IFA. **(C)** The sera and **(B)** spleen homogenates of the ducklings at 0–72 hpi were prepared for detection the viral titer by TCID_50_ assay in DEF cells. **(D)** Five randomly selected ducklings in infected group and control group, respectively, were monitored rectal temperatures daily.

The inoculated ducklings began to display clinical symptoms on day 4 post-infection (dpi) characterized by anorexia and weight loss. Two infected ducklings got slight legs paralysis and were reluctant to move on 4–7 dpi. Infected ducklings developed a fever, showing that rectal temperatures increased from day 3 to day 7 post-inoculation and then gradually returned to normal from 8 to 10 dpi (Figure 1D). At necropsy, no lesions were observed except swollen spleens on 1–3 dpi. No duckling was died throughout the monitoring period. Later on IFA test was carried out to detect viral antigens. We observed that viral antigens were detectable in the spleen, kidney, and bursa of fabricius tissues, with a higher viral burden in the spleens (Figures 1A,B). Surprisingly, viremia (10^1.25^ TCID_50_/0.1mL) was observed in serum samples from 1 to 3 dpi (Figure 1C). No virus was detected in other tissues including brain, pancreas, and liver. All the ducklings in control group were healthy and no viral antigen was detected. These findings indicate that ATMUV exhibits mild pathogenicity in young ducklings following intramuscular injection.

### Expression of IFNS and IFITM family is obviously upregulated in duckling in response to ATMUV infection

**Figure 2:**
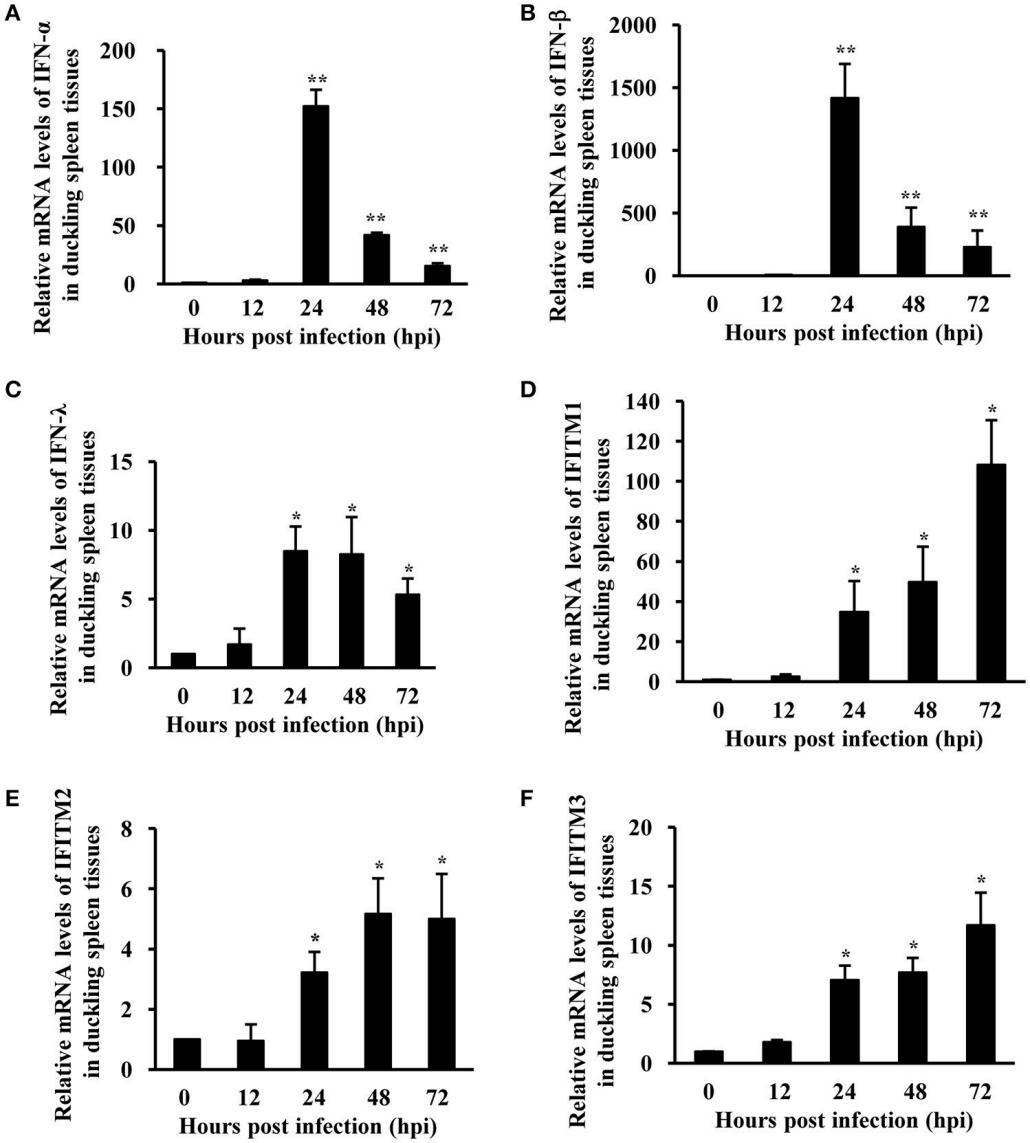
**Expression of IFNs and IFITM family is strongly upregulated in duck in response to ATMUV infection**. Twenty five 7-day-old healthy mule ducklings were challenged by intramuscular injection with 4.0 × 10^5^ ELD_50_ of ATMUV in a volume of 0.4 mL per duckling. Three randomly selected ducklings were sacrificed at 0, 12, 24, 48, and 72 hpi, respectively. Their spleen tissues were collected for examination of IFN-α **(A)**, IFN-β **(B)**, IFN-λ **(C)**, and IFITM1, 2, 3 **(D–F)** mRNA expression using qRT-PCR. The mRNA levels were normalized to the endogenous β-actin level and the expression at 0 hpi was set to 1.0. Expression at 12–72 hpi was compared to its expression at 0 hpi. Each sample was analyzed in triplicate and the results are depicted as means ± SD (*n* = 3). Statistical significance was determined by one tail Student's *t*-test analysis. ^*^P < 0.05, ^**^P < 0.01.

Our previous studies had shown that ATMUV infection effectively triggers the host innate immune response, including robust upregulation of type I and type III IFNs and some critical ISGs in chicken and CEF (Chen et al., 2016a). However, innate immune response against ATMUV infection in duck still remains to be determined. Importantly, the role of IFITMs in defending against ATMUV infection has not been well-defined in any avian species. Here, we examined the expression profile of IFNs and IFITMs in parenchymal organs of ducks infected with ATMUV using qRT-PCR and RT-PCR analysis. The results showed that ATMUV has higher replication in duckling spleen tissues, as indicated by obvious expression of viral E gene (Figure 1A; Supplementary Figure 1). Remarkably, the mRNA levels of type I and type III IFNs were gradually elevated and reached their maximum value on 24 hpi and then declined gradually. In particular, IFN-α and IFN-β were greatly upregulated by 152- and 1,415-fold at 24 hpi, respectively (Figures 2A,B). The expression of IFN-λ was modestly upregulated by 8.5-fold at 24 hpi (Figure 2C). The expression of duck IFITM1, 2, and 3 were gradually induced and reached their maximum value at 72 hpi. Strikingly, expression of duck IFITM1 was markedly increased by over 100-folds at 72 hpi (Figures 2D–F). These observations were further confirmed by RT-PCR analysis (Supplementary Figure 1). Moreover, we examined the mRNA expression profile of IFNs and IFITMs in kidney and bursa of fabricius tissues. We found that in kidneys, the mRNA levels of IFNs and IFITMs were modestly upregulated until 48 hpi and then declined (Supplementary Figure 2). IFNs and IFITMs expressions in bursa of fabricius were slightly increased with the highest levels at 12 hpi and then declined (Supplementary Figure 3). Taken together, these data indicate that ATMUV infection can trigger innate immune response in ducklings, including upregulation of IFNs and IFITMs.

### IFITMs are significantly induced in DEF and DF-1 cells after ATMUV infection or treatment with IFN

**Figure 3:**
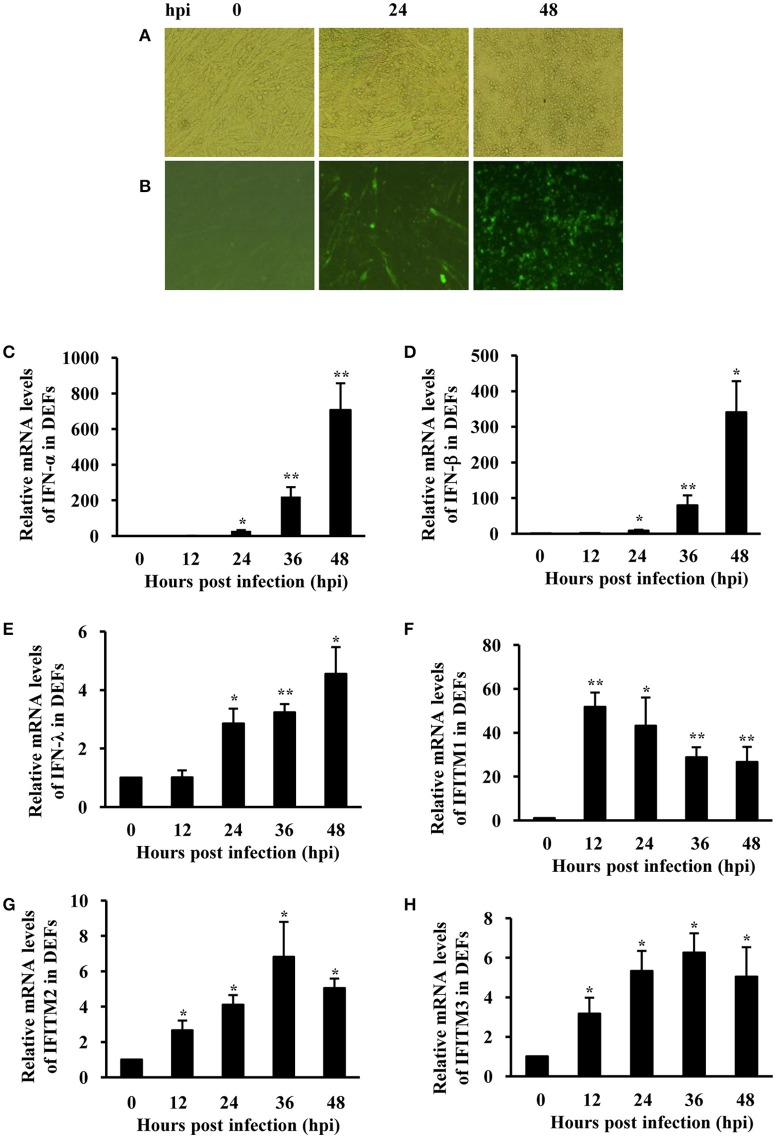
**IFNs and IFITMs are significantly induced in DEFs after ATMUV infection**. DEFs were infected with or without ATMUV at a MOI of 1.0 and harvested at 0, 12, 24, 36, and 48 hpi, respectively. **(A)** CPE feature was recorded at 0, 24, and 48 hpi. **(B)** Viral antigens were detected by IFA in DEF cells at 0, 24, and 48 hpi. qRT-PCR analysis was performed to examine the mRNA expression of duck type I and type III IFNs **(C–E)** and IFITM1, 2, 3 **(F–H)**. The mRNA levels were normalized to the endogenous β-actin level and the expression at 0 hpi was set to 1.0. Expression at 12–48 hpi was compared to its expression at 0 hpi. Plotted are the average levels from three independent experiments with three replicates per experiment (means ± *SD*). Statistical significance was determined by one tail Student's *t*-test analysis. ^*^P < 0.05, ^**^P < 0.01.

To confirm duck innate immune response induced by ATMUV, we further determined the expression of type I and type III IFNs and IFITMs following ATMUV infection *in vitro*. For this, DEFs were prepared and infected with ATMUV, and the mRNA expression of particular IFNs and IFITMs were then analyzed using qRT-PCR. ATMUV replicated well in DEFs with an obvious cytopathic effect (CPE) characterized by cell shrinking, rounding and detachment at 48 hpi (Figure 3A). Analysis of IFA showed that viral antigens were detected in DEF cells at 24–48 hpi, indicating that DEF cells could be infected by the virus (Figure 3B). The mRNA levels of IFN-α, IFN-β, and IFN-λ were significantly elevated in ATMUV infected DEFs during ATMUV infection as compared to mock-treated control. Expression of IFN-α and IFN-β was increased by about 700- and 340-fold at 48 hpi, respectively (Figures 3C–E). IFN-λ expression was also significantly induced with a highest 4.5-fold at 48 hpi. These data were consistent with the *in vivo* studies presented above. Strikingly, duck IFITM1 was greatly induced and reached their maximum value (52-fold) at 12 hpi and then declined gradually (Figure 3F). Expression of duck IFITM2 and IFITM3 were enhanced modestly and reached their maximal levels by 6.8- and 6.3-fold at 36 hpi, respectively (Figures 3G,H). These results were further confirmed by RT-PCR analysis (Supplementary Figure 4). Taken together, these data reveal that innate immune response is triggered in DEFs infected with ATMUV, as indicated by significant upregulation of IFNs and IFITMs.

**Figure 4:**
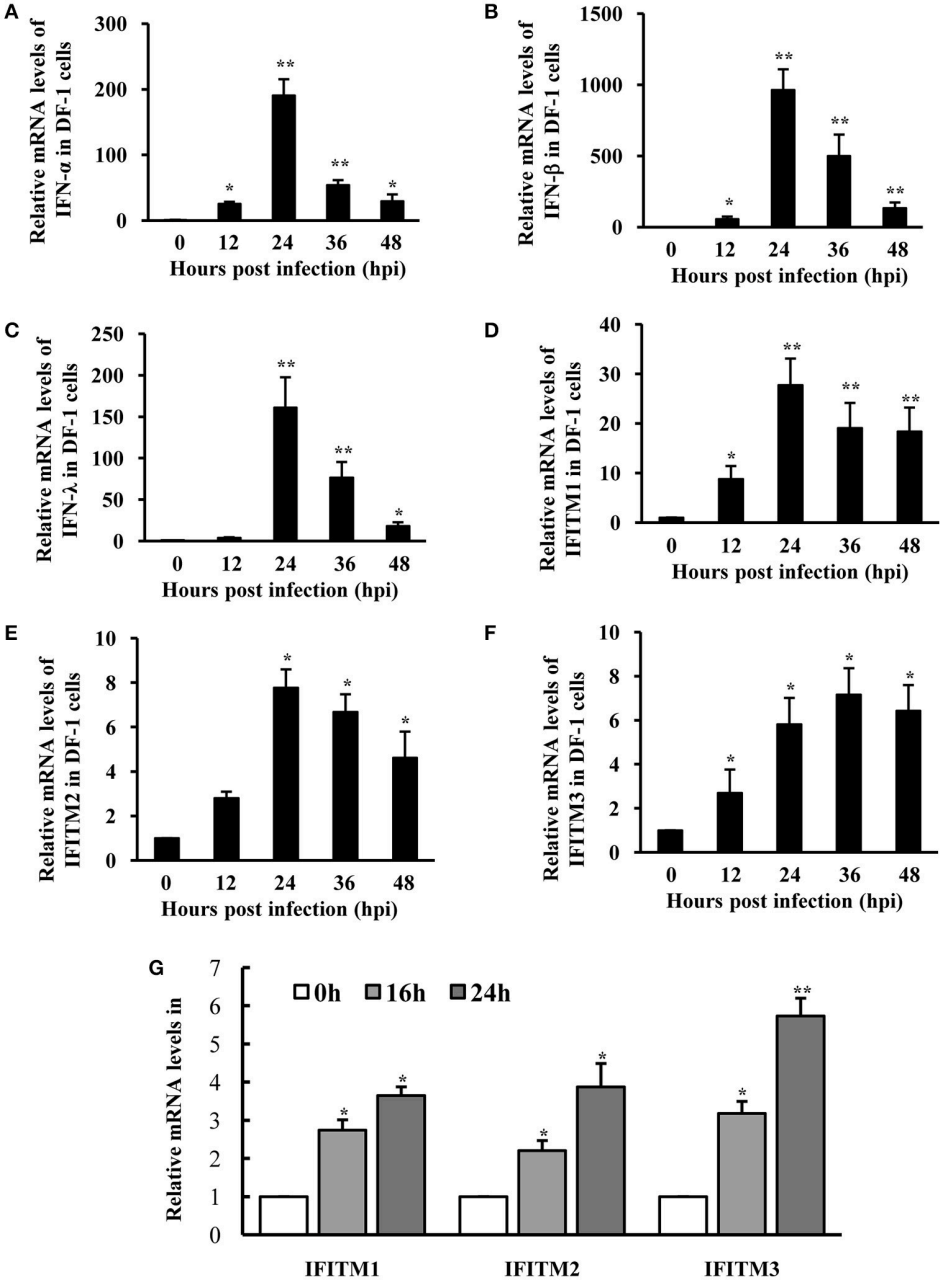
**IFITMs are significantly induced in DF-1 cells after ATMUV infection or treatment with IFN**. DF-1 cells were infected with or without ATMUV at a MOI of 1.0 and harvested at 0, 12, 24, 36, and 48 hpi, respectively. qRT-PCR was performed to determine the relative mRNA expression of indicated IFNs **(A–C)** and IFITM **(D–F)** genes compared with that at 0 hpi. **(G)** DF-1 cells were treated with chicken IFNs (500 IU/mL) for 0, 16, or 24 h. qRT-PCR was performed to determine the relative mRNA expression of IFITM genes compared with that without IFN treatment. The mRNA levels were normalized to the endogenous β-actin level. Plotted are the average levels from three independent experiments with three replicates per experiment (means ± *SD*). Statistical significance was determined by one tail Student's *t*-test analysis. ^*^P < 0.05, ^**^P < 0.01.

Because DEF cells are hard to survive after several passages, we employed chicken DF-1 cell line as a model system to further investigate the interaction between host innate immune system and ATMUV, and the role of IFITMs in defense against viral infection. As expected, mRNA levels of IFN-α, IFN-β, and IFN-λ were greatly elevated by ATMUV infection and reached the maximal levels at 24 hpi and then declined gradually as compared to mock treatment (Figures 4A–C). Similarly, expression of chicken IFITM1 (chIFITM1) was highly upregulated by 28-fold at 24 hpi. Expression of chicken IFITM2 (chIFITM2) and IFITM3 (chIFITM3) was also significantly increased in response to ATMUV infection (Figures 4D–F). These observations were further confirmed by RT-PCR analysis (Supplementary Figure 5).

Since ATMUV infection induced significant upregulation of IFNs and IFITMs *in vivo* and *in vitro*, we asked whether increased IFITM expression was stimulated by ATMUV-induced IFNs in DF-1 cells. To this end, DF-1 cells were treated with chicken IFNs (500 IU/mL) and mRNA levels of IFITMs were examined by qRT-PCR. Indeed, we found that expression of IFITM1, 2, 3 was significantly upregulated in DF-1 cells after treatment with chicken IFNs (Figure 4G).

### Knockdown of endogenous IFITM1 and IFITM3 significantly promotes ATMUV replication in DF-1 cells

**Figure 5:**
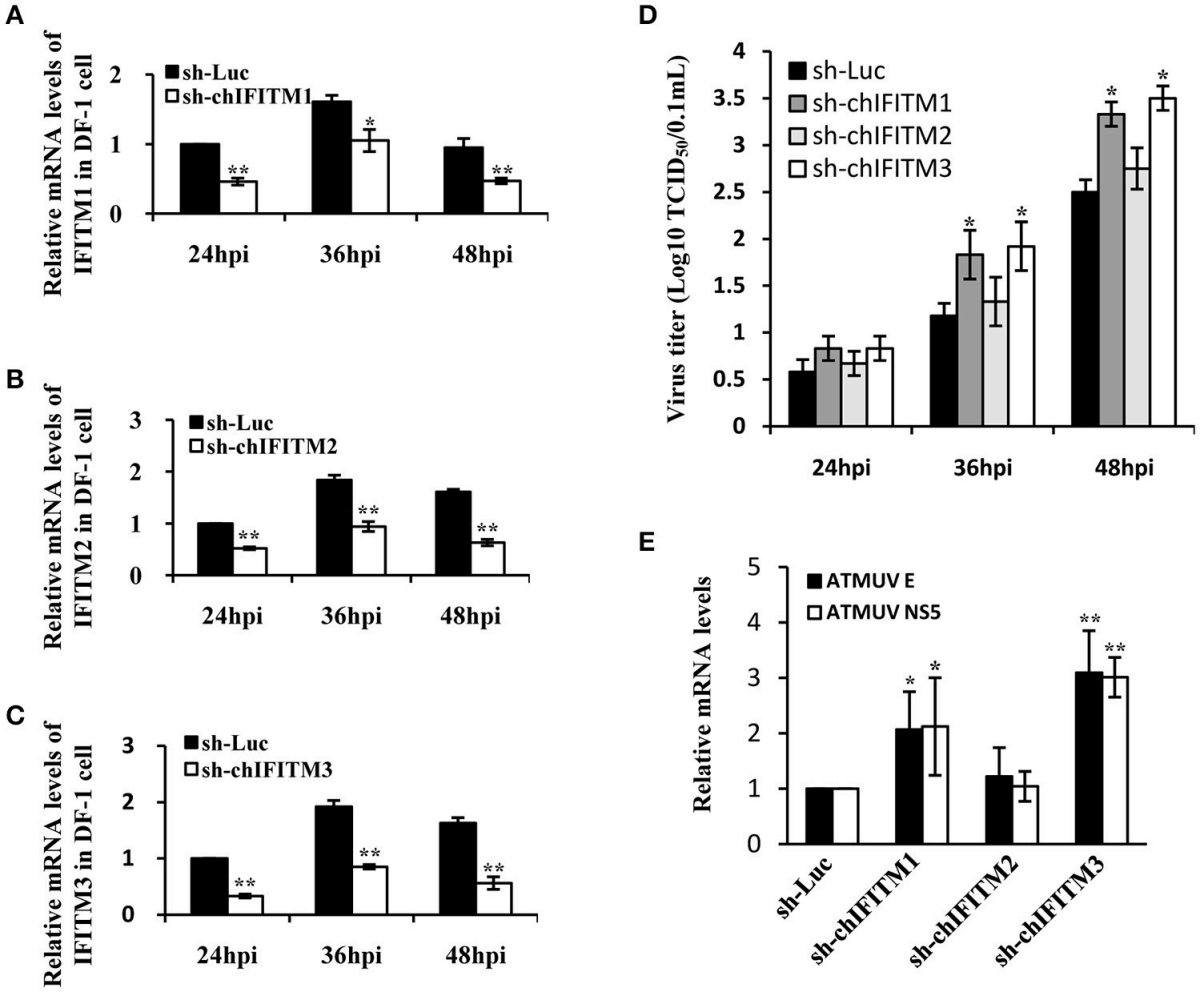
**Knockdown of endogenous IFITM1 and IFITM3 significantly promotes ATMUV replication in DF-1 cells**. DF-1 cell lines stably expressing specific shRNAs targeting chicken IFITM1, 2, 3 or luciferase control were infected with or without ATMUV at a MOI of 1.0 and harvested at indicated time points of 24, 36, and 48 hpi. **(A–C)** qRT-PCR was performed to measure interference efficiency of the shRNAs. **(D)** Viral load in cell culture supernatants was titrated via TCID_50_ assay in DEF cells. **(E)** DF-1 cell lines expressing specific IFITM-shRNA were infected with ATMUV and harvested at 36 hpi, followed by qRT-PCR assay to detect mRNA expression of viral genes. The mRNA levels were normalized to the endogenous β-actin level and the expression in ATMUV infected DF-1 cell line expressing luc-shRNA control was set to 1.0. mRNA expression in DF-1 cells expressing specific IFITM-shRNA was compared to that in control cells expressing luc-shRNA. Plotted are the average results from three independent experiments (means ± *SD*). Statistical significance was determined by one tail Student's *t*-test analysis. ^*^P < 0.05, ^**^P < 0.01.

Results presented above revealed that ATMUV infection effectively induced the expression of particular type I, type III IFNs, and IFITMs *in vivo* and *in vitro*. Furthermore, we tested that whether these IFITMs functioned in defense against the virus infection. For this, we generated stable DF-1 cell lines expressing specific shRNAs targeting IFITM1, IFITM2, IFITM3, or luciferase control, respectively. These cells were then infected with ATMUV and harvested at indicated times (24, 36, and 48 hpi). Interference efficiency of the shRNAs was examined by qRT-PCR and viral load in cell culture supernatants was titrated via TCID_50_ assay. Compared with the control shRNA targeting luciferase, the specific shRNAs caused decreased expression of IFITM1, IFITM2, or IFITM3 after ATMUV infection, respectively (Figures 5A–C). Interestingly, silencing endogenous IFITM1 or IFITM3 resulted in significant increase in viral load, as evidenced by higher viral titers in culture supernatants from the IFITM1 or IFITM3 knockdown cells than those in supernatants of luciferase control cells (Figure 5D). However, knockdown of IFITM2 only slightly enhanced ATMUV replication (Figure 5D). To confirm these findings, DF-1 cell lines expressing specific shRNA targeting each of these IFITMs were infected with ATMUV and harvested at 36 hpi, followed by qRT-PCR assay to detect mRNA expression of viral genes. Consistent with the results from TCID_50_ assay, data of qRT-PCR showed that mRNA expression of viral envelope and NS5 genes was clearly increased in IFITM1 or IFITM3 knockdown cells as compared to the control cells. However, no significant difference was observed in viral load between IFITM2 knockdown and luciferase control cells after infection with ATMUV (Figure 5E). This data suggests that disrupting the endogenous expression of chicken IFITM1 and IFITM3 but not IFITM2 can strongly enhance the ATMUV replication in DF-1 cells.

### Overexpression of chicken or duck IFITM1 and IFITM3 can restrict ATMUV infection *in vitro*

**Figure 6:**
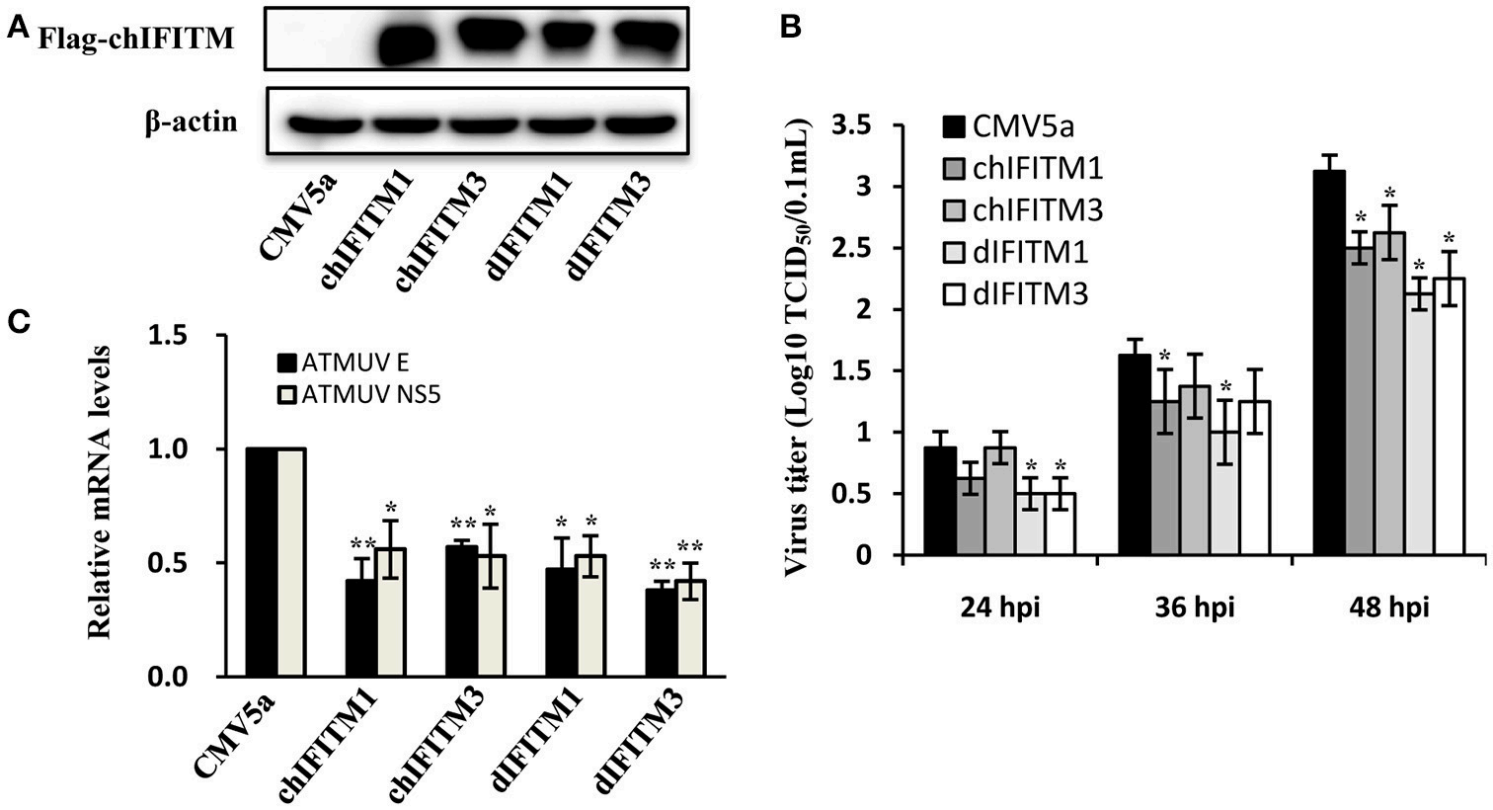
**Overexpression of chicken or duck IFITM1 and IFITM3 restricts ATMUV infection ***in vitro*****. DF-1 cells stably expressing chicken or duck IFITM1, 3, or empty vector were infected with ATMUV at a MOI of 1.0 and harvested at indicated time points (24, 36, and 48 hpi). **(A)** The forced expression of IFITMs in DF-1 cells was examined by Western blotting using an anti-flag antibody at 36 hpi. **(B)** The viral titers in cell culture supernatants were determined by TCID_50_ assay in DEF cells. **(C)** The mRNA expression of viral genes was examined by qRT-PCR at 36 hpi. The mRNA levels were normalized to the endogenous β-actin level and the expression in ATMUV infected DF-1 cells expressing empty vector was set to 1.0. The mRNA expression of viral genes in DF-1 cells expressing exogenous IFITMs was compared to that in control cells containing empty vector. Plotted are the average results from three independent experiments (means ± *SD*). Statistical significance was determined by one tail Student's *t*-test analysis. ^*^P < 0.05, ^**^P < 0.01.

Since silencing IFITM1 and IFITM3 could increase the susceptibility of DF-1 cell to ATMUV infection, we further investigated whether forced expression of IFITMs could inhibit ATMUV replication. Thus, we cloned chicken and duck IFITM1 and IFITM3, and their cDNA sequences were analyzed and deposited in the Genbank database. DF-1 cells were then transiently transfected with constructs expressing chicken or duck IFITM1 and IFITM3 or empty vector, respectively and challenged with ATMUV. Virus titers in cell culture supernatants were determined by TCID_50_ assay at 24, 36, and 48 hpi. The forced expression of IFITMs in DF-1 cells was examined by Western blotting. As shown in Figure 6A, flag-tagged IFITM proteins were expressed well in DF-1 cells (Figure 6A). We observed that overexpression of chicken or duck IFITM1 and IFITM3 obviously suppressed ATMUV replication in DF-1 cells, as indicated by lower TCID_50_ titers in culture supernatants from the IFITM-overexpressing cells than those in control cells (Figure 6B). To further confirm this observation, the DF-1 cells were infected with ATMUV and harvested at 36 hpi, followed by qRT-PCR to examine the viral gene expression. As compared with control cells containing empty vector, overexpression of chicken or duck, IFITM1 or IFITM3 in DF-1 cells reduced mRNA expression of ATMUV envelope gene by average of 58, 43, 53, 62%, respectively (Figure 6C). These data suggest that avian IFITM1 or IFITM3 could strongly interfere with ATMUV infection.

### Analysis of chicken and duck IFITM1 and IFITM3 proteins

**Figure 7:**
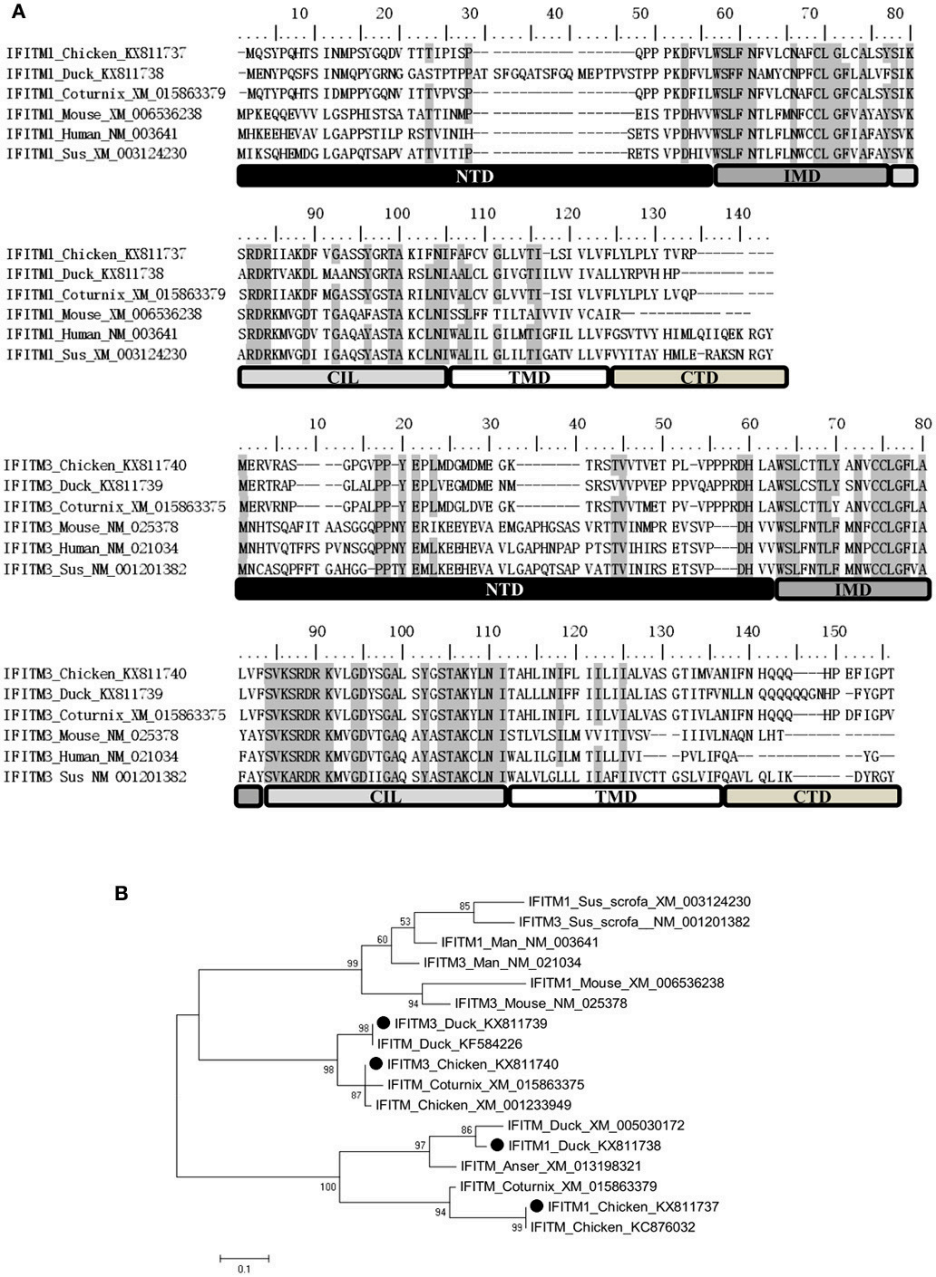
**Analysis of chicken and duck IFITM1 and IFITM3 proteins. (A)** IFITM sequences were aligned using MUSCLE software. Five domains in each sequence alignment were highlighted with different colors: N-terminal domain (NTD), intramembrane domain (IMD), conserved intracellular loop (CIL), transmembrane domain (TMD), and C-terminal domain (CTD). Gray background indicated amino acid identity. **(B)** A maximum-likelihood tree was generated between chicken/duck IFITM1, 3 amino acid sequences, and other species using MEGA 5.0 software. Bootstrap analysis was performed with 1000 trials. Black circle (•): chicken/duck IFITM1, 3 amino acid sequences deposited in the Genbank database with accession numbers by our lab.

Our findings revealed that avian IFITM1 and IFITM3 were involved in cellular restriction of ATMUV infection. Next, we analyzed chicken (DF-1 cell origin) and duck (duck embryo fibroblasts origin) IFITM1 and IFITM3 genes repertoire. The cDNA sequences had been submitted in the Genbank database (accession numbers: chIFITM1, KX811737; chIFITM3, KX811740; dIFITM1, KX811738; dIFITM3, KX811739). BLASTX search showed that the chIFITM1 (KX811737) shared 100, 83.19, 45.11, 34.4, 30.7, and 35.48% amino acid identity to IFITM1 protein of chicken (KC876032.1), coturnix (XM_015863379.1), duck (KX811738), human (AK290480.1), mouse (XM_006536238.1), and sus scrofa (XM_003124230.2), respectively. chIFITM3 (KX811740) shared 99.27, 90.51, 75.18, 31.54, 32.21, and 33.78% amino acid identity to IFITM3 protein of chicken (XM001233949.4), coturnix (XM_015863375.1), duck (KX811739), human (BC070243.1), mouse (NM-025378.2), and sus scrofa (NM_001201382.1), respectively. Two putative transmembrane domains were obtained by using transmembrane prediction programs TMHMM 2 (http://www.cbs.dtu.dk/services/TMHMM/) and TMpred (http://www.ch.embnet.org/software/TMPRED_form.html). Chicken and duck IFITM1 and IFITM3 proteins could be divided into five domains reflecting their hydrophobicity and conservation as identified in other species (Figure 7A), including a variable, hydrophobic N-terminal domain (NTD), a conserved hydrophobic intramembrane domain (IMD), a conserved intracellular loop (CIL), a variable, hydrophobic transmembrane domain (TMD), and a short, highly variable C-terminal domain (CTD). Although significant divergence is seen between avian and mammalian IFITM1 and IFITM3 sequences, several residues are conserved and important for antiviral function, including important amino acids (Y20, C74, C75, K86, K91, and K107) in IFITM3 (Smith et al., 2013; Blyth et al., 2015). Phylogenetic analysis exhibited that avian IFITM1 and IFITM3 are distinct from those of mammalian species, but avian IFITM3 is more conserved than avian IFITM1 (Figure 7B).

## Discussion

ATMUV is a newly emerging member of the Ntaya virus group within the genus of *Flavivirus*, causing severe egg-drop syndrome and neurological disease in domestic poultry (Liu et al., 2013; Homonnay et al., 2014; Thontiravong et al., 2015). On the basis of typical clinical symptoms, ATMUV was initially described as duck egg drop syndrome virus (DEDSV). However, this virus exhibits a wide range of pathogenicity to avian species including ducks, chicken, geese, house sparrows, and racing pigeon, and its genome is closely related to Tembusu virus strains. Flavivirus isolates with a more than 84% nucleotide sequence homology in the NS5 region are considered to be the same species (Kuno et al., 1998). So we propose to name this novel flavivirus as “Avian Temubsu virus, ATMUV.”

Till now, there is no effective method for its prevention except vaccine. The previous studies have shown that ATMUV infection induced an effective antiviral immunity through MDA5 and TLR3-dependent signaling pathways (Li et al., 2015; Chen et al., 2016a; Fu et al., 2016). However, our understanding of the molecular and cellular basis of interaction between the virus and host antiviral immune system is very limited. In this study, we examined the expression profile of key IFNs and IFITMs following ATMUV infection *in vivo* and *in vitro*. We found type I, type III IFNs, and immune-related IFITMs, including IFITM1, IFITM2, and IFITM3 were significantly upregulated in response to ATMUV infection. Strikingly, the spleen tissue had a high viral load and showed strong innate immune response as compared to other organs. IFN-α/β expression levels in spleen were increased significantly at 24 hpi and decreased after this time point, and the mRNA levels of IFITM1 and IFITM2 were also upregulated. Similar results were shown in kidney and bursa of fabricius tissues in infected animals. Although IFN expression in DEFs was triggered and reached the maximum value at 48 hpi, DEFs displayed an apparent CPE by ATMUV infection at this time point. These data suggest that host innate immune response is triggered by ATMUV, but the virus has developed multiple strategies to evade host antiviral immunity. In a recent study, Wang and his co-workers found that ATMUV NS1 could markedly suppress virus-induced IFN-β expression by inhibiting RLR receptor signaling (Wang J. et al., 2016). Most flaviviruses are potent in blocking the JAK-STAT pathway to evade the antiviral effects of the host innate immune system (Heim et al., 1999; Basu et al., 2001; Lin et al., 2004; Green et al., 2014). Thus, further studies are needed to address the precise mechanisms by which ATMUV circumvents the host innate immune response.

Type I and Type III IFNs bind to their receptors, which stimulates the JAK-STAT pathway that triggers various ISG expression. Expression of IFITMs is strongly induced by IFNs (Smith et al., 2013). Consistent with previous studies (Smith et al., 2013), our data showed that stimulation of DF-1 cells with chicken IFNs significantly enhanced the expression of IFITMs. Interestingly, we observed that IFITM1 in ATMUV infected DEFs had a faster kinetic response than ATMUV-induced IFNs. Thus, we assessed whether IFITMs expression is totally IFN-dependent. For this, siRNA specifically targeting chicken interferon alpha/beta receptor 1 (chIFNAR1-siRNA) and negative control siRNA (NC-siRNA) were employed in this study. 100 nM chIFNAR1-siRNA or NC-siRNA was transfected into DF-1 cell. Twenty-four hours post-transfection, the cells were infected with ATMUV and harvested at 24 hpi. Interestingly, disruption of endogenous IFNAR1 by siRNA greatly reduced IFITM2 mRNA expression, but only caused modest downregulation of IFITM1 and IFITM3 (Supplementary Figure 6). The data suggest that chIFITM2 may not share the same IFN-dependent pathways with chIFITM1 and chIFITM3. Previous study has found that murine IFITM3 expression was not only induced by increased expression of type I or II IFNs, but also upregulated by other cytokines such as IL-6 (Bailey et al., 2012). Other groups also found that expression of IFI-56K, IFI-54K, and ISG56 was induced directly by virus and then triggered secondarily through virus-induced IFNs (Wathelet et al., 1988; Holzinger et al., 2007). These findings indicate that multiple signaling pathways might be involved in regulation of IFITM expression during the viral infection.

IFITMs serve as critical effector molecules in host innate immune system and effectively restrict a wide range of pathogenic viruses, such as dengue virus, influenza A virus, Hepatitis C Virus, West Nile Virus, and HIV-1 (Brass et al., 2009; Wilkins et al., 2013; Yu et al., 2015). In order to survive, some viruses such as arenavirus and foot-and-mouth disease virus have evolved multiple strategies to evade the antiviral effects of IFITMs (Bailey et al., 2014; Zhang et al., 2016). Furthermore, human cytomegalovirus could exploit IFITMs to facilitate morphogenesis of virion assembly compartment (Xie et al., 2014). To investigate the role of IFITM proteins in restricting ATMUV replication, we generated DF-1 cell lines stably disrupting chicken IFITM1, IFITM2, or IFITM3 expression. Indeed, disruption of endogenous IFITM1 and IFITM3 by shRNA greatly enhanced ATMUV infection. Furthermore, ectopic expression of chicken and duck IFITM1 or IFITM3 markedly inhibited ATMUV replication. Interestingly, chIFITM2 was significantly upregulated after ATMUV infection *in vitro*, but we found that altering IFITM2 expression had mild effect on ATMUV infection. A previous report showed that duck IFITM1 was greatly upregulated after highly pathogenic IAV infection but its antiviral activity was low *in vitro* (Blyth et al., 2015). The differences in viral entry mechanisms of IAV and ATMUV in the different types of host cells may account for the differential antiviral activity of IFITM proteins.

Five IFITM proteins have been identified in duck and chicken species including IFITM1, IFITM2, IFITM3, IFITM5, and IFITM10. IFITM1, IFITM2, and IFITM3 belong to the immune-related clade, whereas non-immune IFITM5 and IFITM10 make up the two remaining clades (Blyth et al., 2015). In this study, we observed that IFITM1 increase fold was much higher than those of IFITM2 and IFITM3 by *in vivo* and *in vitro* assays. This may be due to high basal expression of IFITM2 and IFITM3 in the cells tested. In addition, we cloned and characterized chicken and duck species of IFITM1 and IFITM3 genes. Despite sharing low amino acid identity between chicken IFITM1 and duck IFITM1 (45.11%) and high amino acid identity between chicken IFITM3 and duck IFITM3 (75.18%), both chicken and duck origin IFITM1 and IFITM3 could effectively restrict ATMUV replication. Certain key amino acids in IMD and CIL domain are conserved in chicken and duck IFITMs, suggesting their importance for functioning of the IFITMs (Smith et al., 2013).

Our previous work demonstrated that pretreatment of cells with IFNs could significantly impair ATMUV replication (Chen et al., 2016a). Our present study further provides the evidence that host IFITM proteins have the ability to control the ATMUV infection and likely restrict the viral reproduction as well. Taken together, ATMUV infection induces host's effective antiviral immune response involving several critical IFNs and IFITMs proteins, which can be useful for developing new antiviral drugs in future. However, further studies are required for better understanding of precise mechanisms underlying the antiviral activity of IFITM proteins.

## Author contributions

SLC performed most of the experiments, collected and analyzed data and wrote the manuscript. LW, LZ, WS, YN, and ZY participated in preliminary data acquisition. JYC contributed to plasmids construction. XZ helped with data analysis. SW and XC contributed to shRNA design and revised the manuscript. JLC and MG revised the manuscript. SYC and JLC conceived of the study, participated in study design and coordination. All authors read and approved the final manuscript.

## Funding

This study was supported by Natural Science Foundation of China (U1405216), the National Basic Research Program (973) of China (2015CB910502), National Key Research and Development Program of China (2016YFD0500205), and Intramural grant of Fujian Agriculture and Forestry University.

### Conflict of interest statement

The authors declare that the research was conducted in the absence of any commercial or financial relationships that could be construed as a potential conflict of interest.
