# Supplementary figures and images for: Avian Interferon-Inducible Transmembrane Protein Family Effectively Restricts Avian Tembusu Virus Infection
Source: Front Microbiol. 2017 Apr 20;8:672. doi: 10.3389/fmicb.2017.00672 (PMC5397487; doi:10.3389/fmicb.2017.00672)

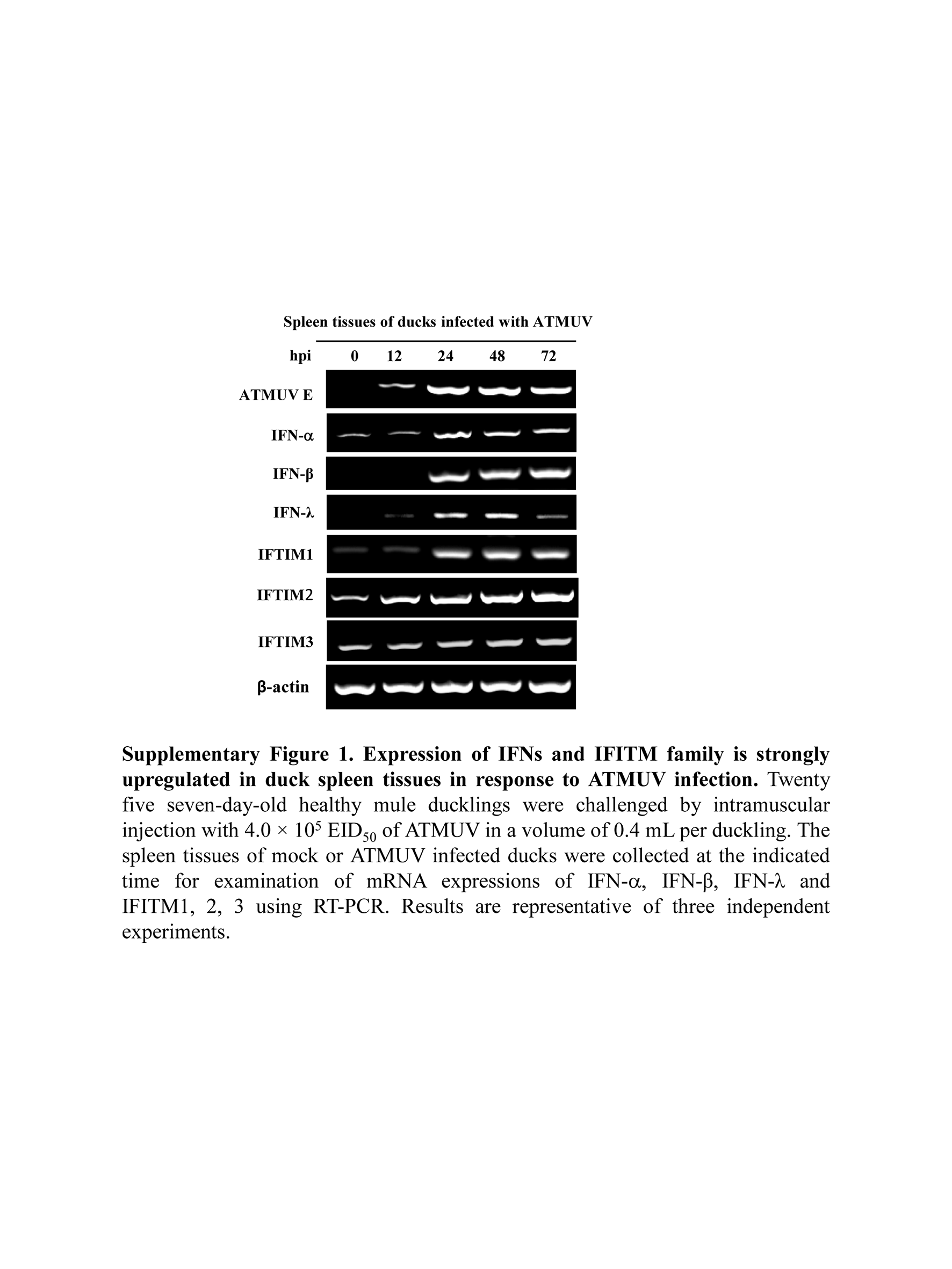

Supplement: Supplementary file 1 [file Image1.TIF]

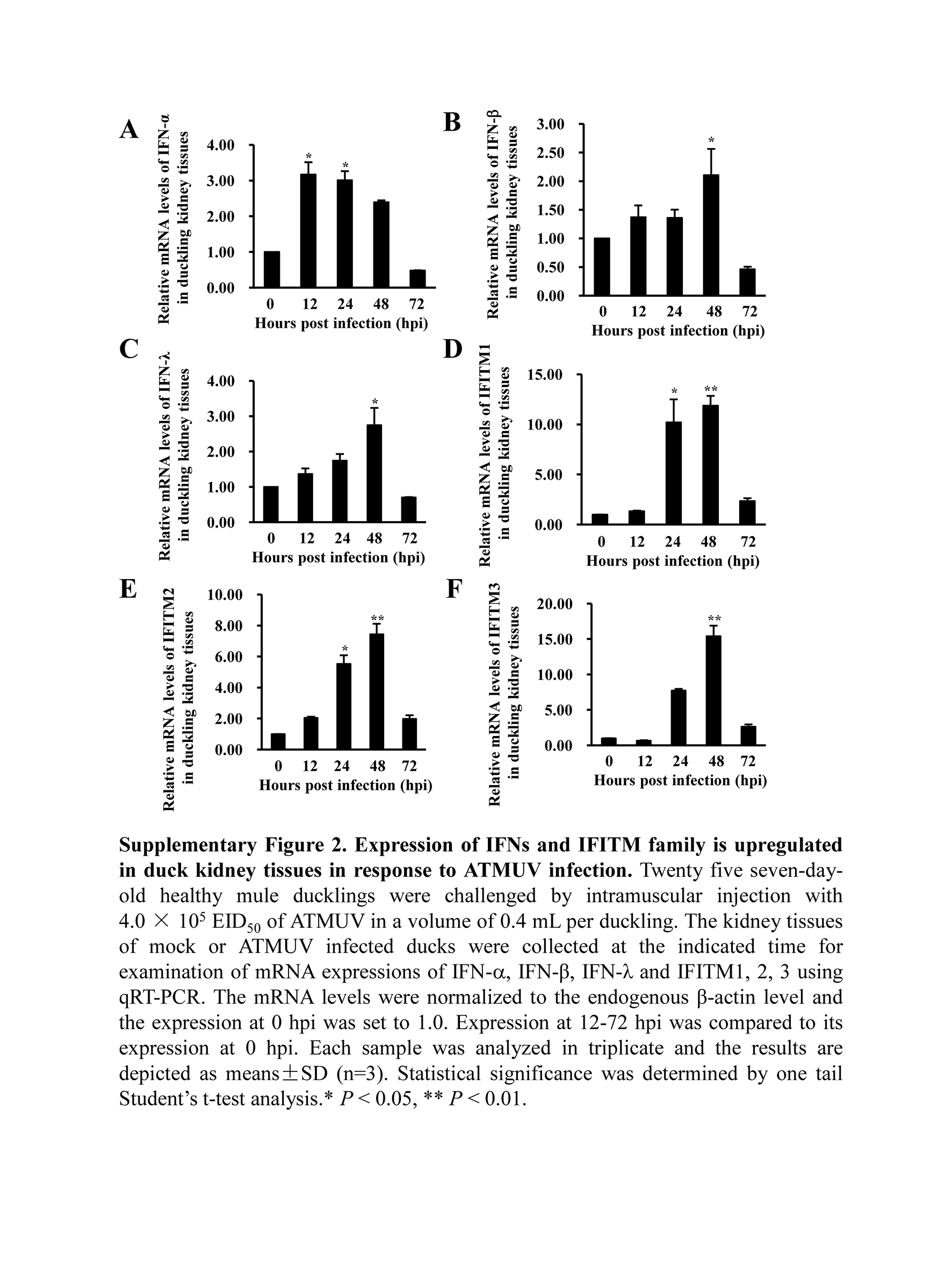

Supplement: Supplementary file 2 [file Image2.TIF]

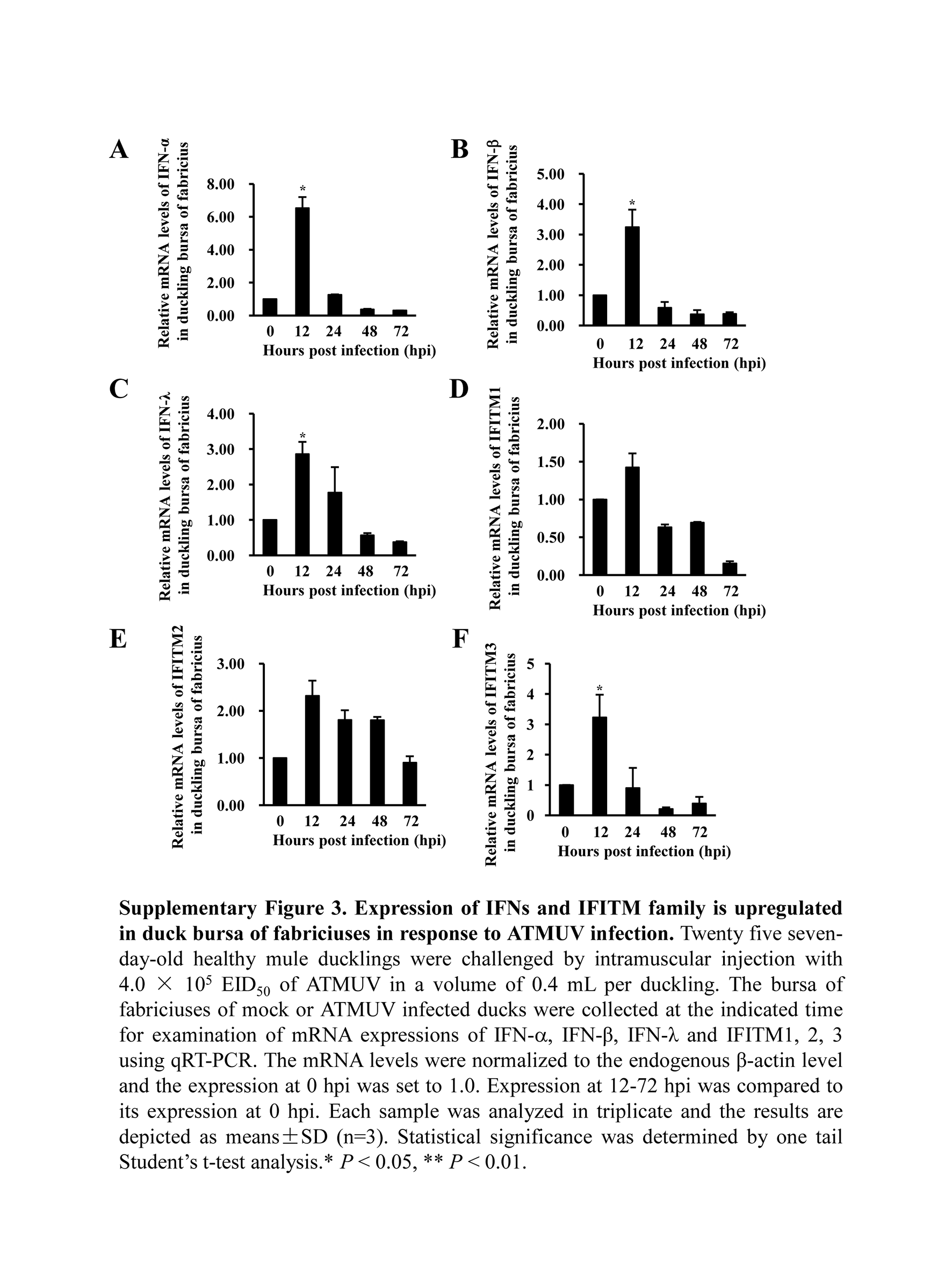

Supplement: Supplementary file 3 [file Image3.TIF]

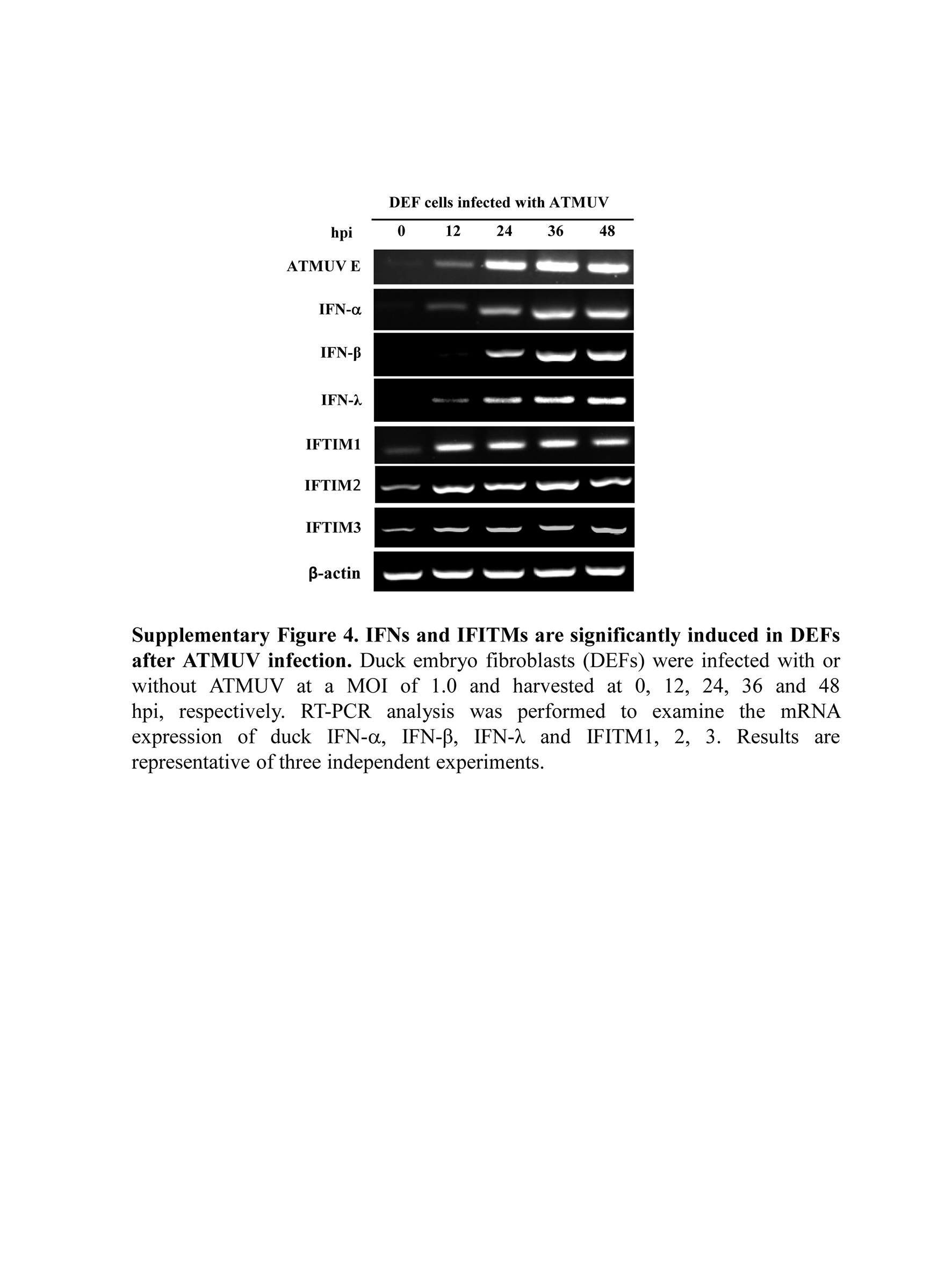

Supplement: Supplementary file 4 [file Image4.TIF]

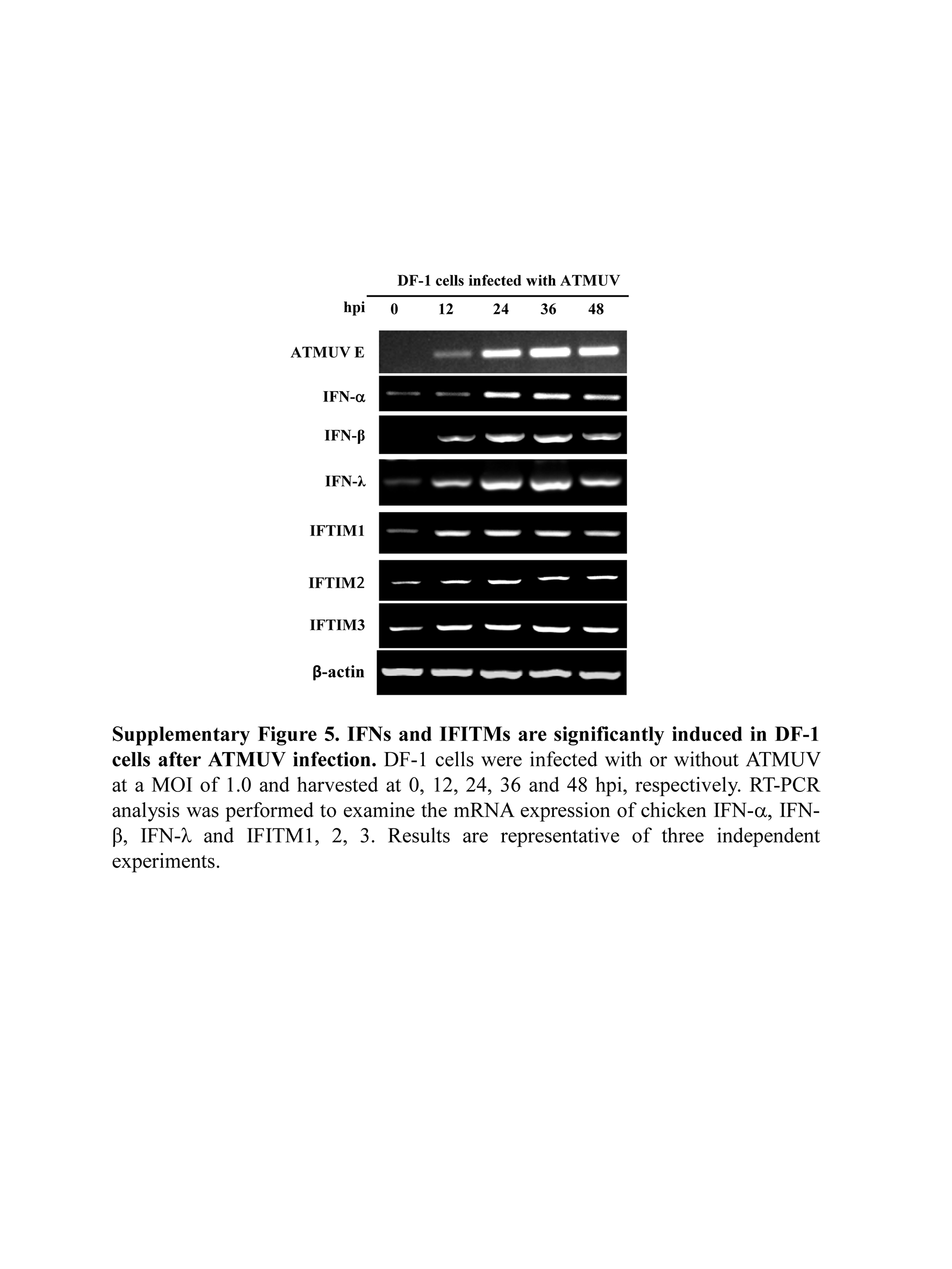

Supplement: Supplementary file 5 [file Image5.TIF]

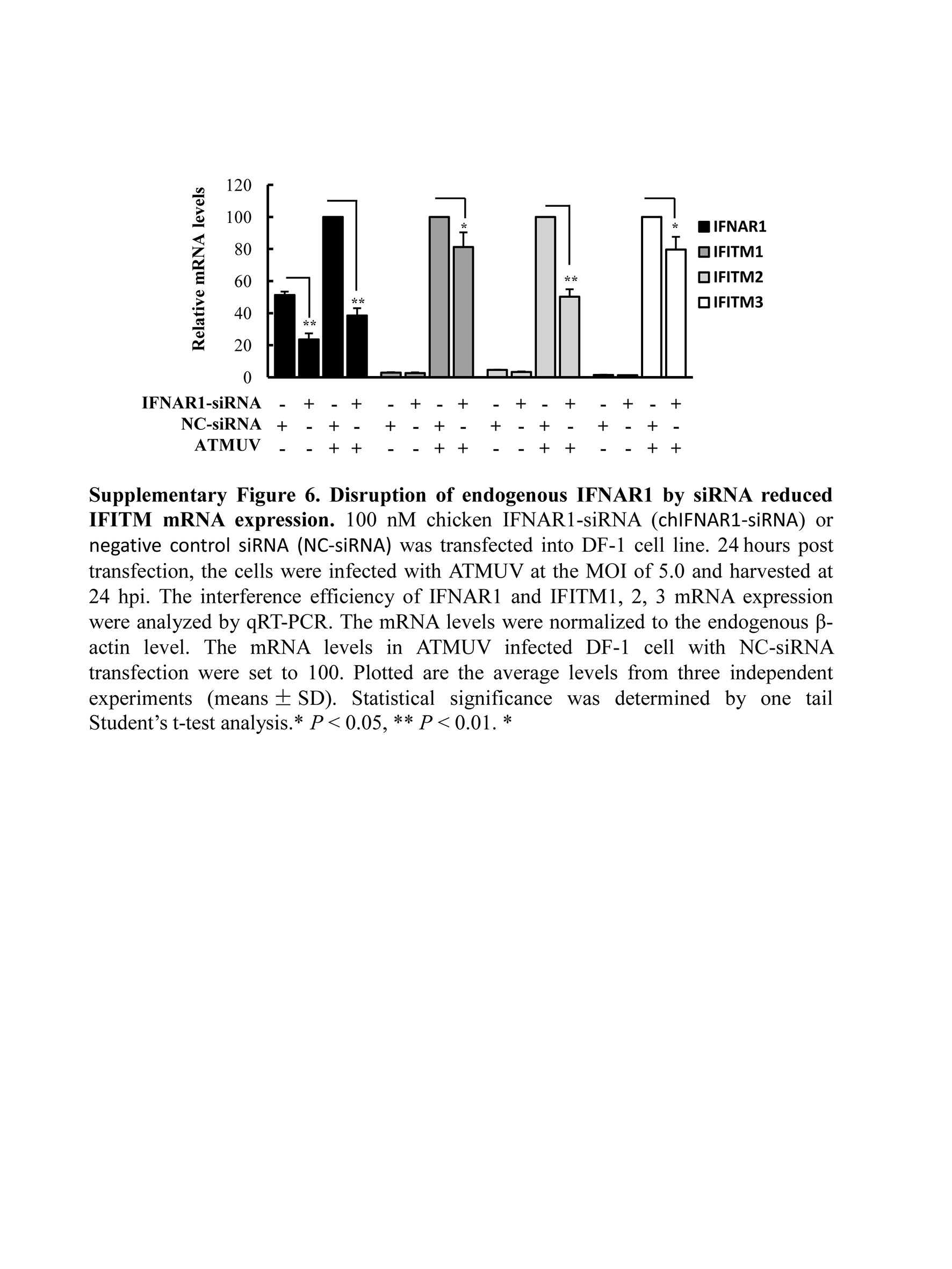

Supplement: Supplementary file 6 [file Image6.TIF]
